# Explanatory models of common mental disorders among South Asians in high-income countries: A systematic review

**DOI:** 10.1177/13634615241296302

**Published:** 2025-01-09

**Authors:** Ruchika Jain, Ritsuko Kakuma, Daisy R. Singla, Kirsty Andresen, Khawater Bahkali, Abhijit Nadkarni

**Affiliations:** 1 Department of Population Health, London School of Hygiene and Tropical Medicine, London, UK; 2 Campbell Family Mental Health Research Institute, Center for Addiction and Mental Health, Toronto, Canada; 3 Department of Psychiatry, Temerty Faculty of Medicine, 7938 University of Toronto, Toronto, Canada; 4 Lunenfeld Tanenbaum Research Institute, 518775 Sinai Health, Toronto, Canada; 5 Department of Non-Communicable Disease Epidemiology, 4906 London School of Hygiene and Tropical Medicine, London, UK; 6 Department of Infectious Disease Epidemiology, 4906 London School of Hygiene and Tropical Medicine, London, UK; 7 Public Health Intelligence, Public Health Authority, Riyadh, Saudi Arabia; 8 Addictions and Related Research Group, Sangath, India

**Keywords:** culture, mental health, qualitative research, explanatory model, South Asian, systematic review

## Abstract

Mental health service use by individuals of South Asian origin living outside of South Asia is influenced by cultural factors such as endorsing psycho-social-spiritual over biological explanations, somatisation, and stigma. The aim of this review is to synthesise the evidence about (a) explanatory models of common mental disorders (CMDs) among people of South Asian origin residing in high-income countries, and (b) their help-seeking for CMDs, including formal and informal care. The systematic review protocol was registered a priori on Prospero (registration number CRD42021287583). We ran extensive searches on explanatory models and help-seeking of people of South Asian origin across five databases (MEDLINE, Embase, Cumulated Index to Nursing and Allied Health (CINAHL), PsycINFO, and Global Health). We extracted the data and conducted a narrative synthesis. We included 33 reports and 29 studies (9,030 participants). The participants in the included studies viewed CMDs through a psychosocial rather than a biological lens (e.g., resulting from family issues vs. neurotransmitters). Causal attributions included life stressors and attitudinal and religious/spiritual factors. Commonly used help-seeking strategies included private coping (i.e., crying or praying), speaking to friends and family, and visiting their General Practitioner. We can conclude that cultural factors play an important role in how South Asian individuals experience and understand CMDs. To cope, they use pluralistic help-seeking strategies. Implications for clinical practice and policy include increasing research on the explanatory models of CMDs, involving family in services, and developing community-based interventions for individuals who do not engage with formal care.

## Background

Common mental disorders (CMDs), which include depression and anxiety disorders, are leading causes of disease burden, with a global prevalence estimate of 29% for adults within their lifetime (Steel et al., 2014). The prevalence of depression ranges from 5% to 44% for first-generation immigrant groups versus 8% to 12% for the general population, and for anxiety it ranges from 4% to 40% in first-generation immigrant groups compared to 5% in the general population (Close et al., 2016). While prevalence estimates of CMDs among South Asian individuals vary (Bhavsar et al., 2021), there is consensus that people of South Asian origin who reside outside of South Asia experience similar or higher rates of CMDs than the native population. For example, in the UK, South Asians have lower levels of psychosis but higher levels of deliberate self-harm and psychological distress than the majority White population (Bhavsar et al., 2021) and in Canada the prevalence of depressive symptoms among South Asians (21%) was double that of the national average (10%) (Lai & Surood, 2013).

However, immigrants are less likely than the general population to access mental health care for reasons such as language, stigma, cultural perception, experiences (i.e., presentation of symptoms), and a limited understanding of a new healthcare system (Lu et al., 2020; Pollard & Howard, 2021). Even if mental health services are accessed, there is a significant delay when compared to the native population (Bhui et al., 2003; Fernando, 2014; Tribe & Marshall, 2020). South Asian immigrants (individuals originating from India, Pakistan, Sri Lanka, or Bangladesh) underuse mental health services in comparison to their White counterparts and other ethnic minority groups (Bowl, 2007; Hussain & Cochrane, 2016; Prajapati & Liebling, 2022). For example, Pakistani and Bangladeshi women in England are less likely to access mental health services than White women (Pakistani OR = 0.23, Bangladeshi OR = 0.25) (Kapadia et al., 2018), and Asian groups in the UK are 38% less likely to have received treatment for a CMD than White groups in the UK (Ahmad et al., 2021).

Meeting the needs of immigrant groups is important considering that the global movement of people, driven by interconnecting social, political, environmental, and economic factors, has increased, particularly from low-income country to high-income country (HIC) settings (Bhugra, 2004; United Nations Department of Economic and Social Affairs, 2020). Particularly, South Asians represent a rapidly growing ethnic group in HICs such as the United States, UK, Canada, and Australia (Prajabati & Liebling, 2022). Besides the challenges of the migration process itself (particularly in cases of forced migration), post-migration acculturative stress, socio-economic-political conditions, familial factors, age of migration, and country of birth (e.g., discrimination, position in society) in the host country may cumulatively contribute to CMDs in immigrants and their subsequent generations (Bhugra, 2004; Crowley, 2022; Karasz, 2005; Lubin & Khandai, 2017).

In addition to systemic barriers, one explanation for poor service use is the explanatory models of illness—defined as “the way people perceive, interpret and respond to [illness]” (Dinos et al., 2017, p. 106). Explanatory models influence whether someone seeks or receives formal or informal help, when they receive help, who they receive help from, and how effective the help is (Kleinman et al., 2006). If a healthcare provider's explanatory model differs from their patient's, providers may face difficulties empathising with their patient, as well as understanding their symbolic language, explanations of illness, and their perspective on the role/responsibility of the healthcare provider (Bhui et al., 2013; Delara, 2016; Gopalkrishnan, 2018). This can lead to misdiagnosis, delayed diagnosis, or referrals to inappropriate services for immigrant and ethnic minority groups who may refer to maintain their cultural and religious perceptions of health (Bowl, 2007; Helman, 1994).

Discounting an individual's explanatory model in a clinical setting contributes to poorer treatment outcomes, particularly for immigrant and ethnic minority groups (Bhui & Bhugra, 2003). Conversely, exploring explanatory models can centre the role of culture in mental health experience and management, allow for a tailored response to an individual's generational status and life context, involve a patient's family in decision-making, reduce stigma, diversify treatment options / sources of help, and ultimately lead to greater and more appropriate use of mental health services (Dinos et al., 2017).

South Asian individuals living in HIC settings often do not perceive available mental health services or mental health prevention initiatives to be culturally appropriate (Bhui & Bhugra, 2002; Bowl, 2007; Islam et al., 2014). In Canada, South Asian individuals with a major depressive disorder had the highest percentage of unmet mental health care need (48%) and perceptions of barriers to mental health service use (33%) compared to eight other ethnic minority groups (Gadalla, 2010; Islam et al., 2014).

This may be partly explained by the overuse of the biomedical model in Western settings, which fails to account for psychological, social, and cultural factors, and emphasises individualism, biological explanations, and pharmacological treatment for mental health conditions (Deacon, 2013). These characteristics are in direct contrast with many South Asian individuals’ explanatory models regarding the cause and appropriate treatment of CMDs, somatic expression of symptoms, and collectivism. These are all directly related to refusal of mental health service use (Antoniades et al., 2017; Beiser et al., 2003; Karasz, 2005; Rastogi et al., 2014).

South Asian individuals in HIC settings understand CMDs through religious (e.g., supernatural forces or God's will), social (e.g., difficult life events), or moral (e.g., weakness) lenses rather than as an illness requiring biomedical intervention (Antoniades et al., 2017; Gilbert et al., 2006; Jacob et al., 1998; Karasz et al., 2013; Rastogi et al., 2014). These explanations link with stigma, which may cause South Asian individuals to believe that seeking formal help could lead to humiliation in their community if their CMD is perceived to result from a character flaw (Karasz et al., 2019). South Asian individuals may therefore prefer to seek help for mental health problems from informal support (such as relying on oneself, family, or faith healers) rather than Western healthcare services (Bradby et al., 2007; Hussain & Cochrane, 2010).

South Asian individuals often express their mental distress as somatic symptoms (Anand & Cochrane, 2005; Gunasinghe et al., 2019; Hussain & Cochrane, 2016; Karasz, 2005). Along with language barriers, this can cause miscommunication between service users and care providers (who largely possess a biomedical view on health and disease such as the body–mind dichotomy) (Bhui et al., 2013).

Individual–collectivist discord is an important cultural factor that plays a role in determining help-seeking (Soorkia et al., 2011). Collectivism is an important aspect of South Asian identity; South Asian individuals often prioritise family over the individual (Masood et al., 2009; Tummala-Narra, 2013). Collectivist values, for both South Asian parents and their children, can inhibit the sharing of mental health concerns if the affected individual does not want to place burden on their family members or is conscious of embarrassing their family in the wider community. If individuals do seek professional help, the care offered could be incompetent if a host country's Western model of medicine does not, for example, consider parent–child relations in immigrant families, where views on family obligation differ from the mainstream culture's ideology (Bismar, 2018).

It is well established in the literature that culture influences how individuals understand, experience, and manage mental health conditions. However, there is yet to be a review on the specific cultural factors that influence mental health in the South Asian diaspora, which can inhibit the delivery of, and access to, culturally informed care for this population. To grow the evidence base, this systematic review aims to synthesise the evidence about: (1) explanatory models of CMDs among people of South Asian origin residing in HICs; and (2) their help-seeking attitudes, intentions, and behaviours towards informal and formal mental health support for CMDs.

## Method

### Design

Systematic review of observational studies. The systematic review protocol was registered a priori on Prospero (registration number CRD42021287583).

### Eligibility criteria

We included peer-reviewed research articles describing observational studies, published in English. We did not have any restrictions on year of publication. We included studies that described the explanatory models of and help-seeking for CMDs by South Asians (from India, Pakistan, Sri Lanka, or Bangladesh) living in HICs as categorised by the World Bank. CMDs were defined as depression and anxiety disorders such as post-traumatic stress disorder, panic disorder, generalised anxiety disorder, obsessive-compulsive disorder, and phobias (Kendrick & Pilling, 2012). Explanatory models were defined as “prior knowledge on the causation, perception, experiences, and traditional belief held by the patients, their caregiver, and the population in general” (Lilhare et al., 2020, p. 327). Help-seeking was defined as “attempts to maximise wellness or to ameliorate, mitigate, or eliminate distress” (Arnault, 2009, p. 2) through informal (personal networks such as friends and family) and formal (including professionals such as General Practitioners (GPs), nurses, psychiatrists, and non-health professionals such as teachers, spiritual and religious leaders, and community workers) support, and self-help (Kim & Lee, 2021).

### Search

We searched electronic databases (MEDLINE, Embase, Cumulated Index to Nursing and Allied Health (CINAHL), PsycINFO, and Global Health) on 6 December 2021, using search terms under the following concepts: South Asian (e.g., Indian, Bengali), CMDs (e.g., depression, anxiety), and explanatory models (e.g., beliefs, attitudes, culture). We updated the search across all databases on 1 February 2023, to find articles published in 2022–2023 to identify recent extant literature. The detailed search strategy is described in the Supplementary Materials. To identify other relevant articles, the bibliographies of the included studies were hand-searched by the primary author (RJ).

### Procedures

After automatic and manual de-duplication in EndNote, the search results were imported into the Rayyan software. Two reviewers (RJ and KB) independently screened the titles and abstracts, and a third reviewer (AN) resolved any conflicts. Two reviewers (RJ and KA) independently did the full-text screening and conflicts were discussed until an agreement was reached. When the search was updated, one reviewer completed the title/abstract screening (RJ) and two reviewers completed the full-text screening (RJ and AN). A data extraction form was designed to extract the data needed to meet the study objectives and covered domains such sample demographics, mental health condition under study, and findings pertaining to explanatory models and help-seeking. The data was first extracted 9 March 2022.

### Quality assessment

Qualitative studies were assessed using the Critical Appraisal Skills Programme (CASP) (CASP, n.d.) which covers the appropriateness of research design and data collection tools, consideration of ethics and the researcher/participant relationship, the rigour of the data analysis, and the overall value of the research. For mixed methods studies, we used the 2018 Mixed Methods Appraisal Tool (MMAT) (Hong et al., 2018) which includes the effectiveness of the sampling strategy and sample, the risk of no-response bias, the appropriateness of the measurements and statistical analysis for quantitative studies, the usefulness of using mixed methods, and the interpretations of the results of the quantitative and qualitative components for mixed methods studies.

### Data synthesis

We conducted a narrative synthesis, which provides a qualitative summary of the findings. Informed by the Economic and Social Research Council's Methods Programme guidance (Popay et al., 2006), we followed the following steps: (1) We conducted preliminary data synthesis using thematic analysis, in which the main, recurrent and/or most important (based on the review question) themes and/or concepts across multiple studies were identified; (2) We examined how the results converged and diverged for participant subgroups (i.e., immigrant generation status, sex, country of origin); and (3) We used the evidence grading systems described above to assess the robustness of the synthesis. We used a flow diagram following the PRISMA guidelines to report the selection process and all results (Page et al., 2021).

### Ethics approval

Ethics approval was not required for this study because it did not involve human participants.

## Results

**Figure 1.**
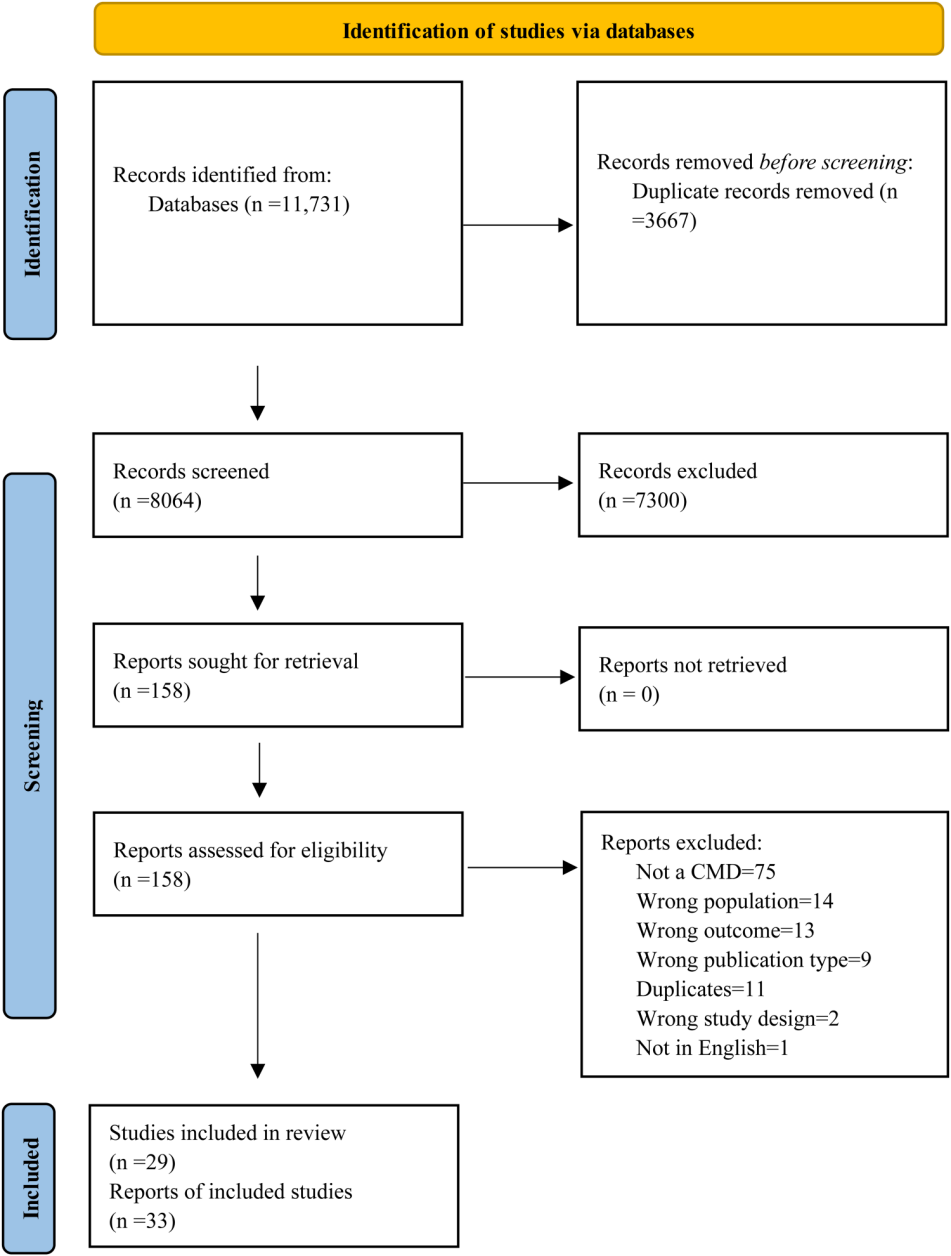
Prisma diagram of identification of included studies in 2022 and 2023.

After removing duplicate articles, we screened 7,445 titles and abstracts, of which 145 records were eligible for full-text screening. A total of 32 articles met our eligibility criteria and were included in the review (Figure 1). When we updated the search in 2023, we screened 619 articles and found seven articles to be eligible for full-text screening. One study was found to be eligible in this round of screening. Finally, we included a total of 33 articles of 29 individual studies (N = 9,030 participants) (Table 1).

**Table 1.** Summary of included studies (n = 29).

Author year	Country setting	Sample size (N) of South Asian participants Mean age (SD) Range	Study design Data collection Method	Explanatory models	Help-seeking
(Furnham & Malik, 1994)	UK University, Community	Native Britons and Asian-Britons (India, Pakistan, Bangladesh) N = 76 *Mean = 32 Range = 17–62	Quantitative Questionnaire	The younger generation of Asian-Britons were more likely to understand depression through a Western lens, similar to their Native Briton peers, than the older generation of Asian-Britons.	The younger-generation Asians preferred to talk to friends, while the older generation preferred to discuss their mental health issues with family.
(Jacob et al., 1998)	UK Primary care	Women of Indian origin N = 100 Mean = 43	Mixed methods Interviews		Those participants who met the GHQ criteria for a CMD were more likely to consult the GP than those who did not. Those with a CMD who did not want to seek medical help were found to have a higher CMD score.
(Bhui et al., 2001, 2002)	UK Primary care	Punjabi and English subjects N = 209 Range = 16–86	Quantitative Survey	The participants with a CMD in the study had symptoms of poor concentration and memory, pain, and depressive ideas. There was no difference between Punjabi people and English people in somatisation. Medical/somatic symptoms were associated with psychological, religious, interpersonal, work, and financial beliefs.	GPs were less likely to diagnose Punjabi cases with depressive ideas with a psychiatric disorder. 46.2% of Punjabi cases did not seek help. Besides the GP, they relied on friends and family. Only 3.9% used religious or traditional strategies.
(Farver et al., 2002)	USA Colleges, universities, high schools	American-born Asian Indian adolescents and one of their immigrant parents N = 360 *Mean = 31 Range = 14–65	Quantitative Questionnaire	Acculturation gaps (between parents and adolescents) was found to be related to anxiety.	
(Commander et al., 2004)	UK Primary care	South Asian and White people with a depressive disorder N = 33 Range = 16–64	Mixed methods Interviews	Most participants (67%) saw their problem as psychosocial.	Most discussed the problem with a relative or friend (70%) and contacted their GP initially (94%), but only 55% discussed their problem with a GP, only 9% saw a mental health professional, and only 9% took a psychiatric medication.
(Burr & Chapman, 2004)	UK Community	Women from South Asian communities N = 46	Qualitative Focus groups (N = 4) and interviews (N = 10)	Participants were willing to use the term ‘depression’ and saw it as an illness but described members of their community as unwilling to. Depression was experienced as many different emotions, and common symptoms were described as being unable to sleep, crying, and fatigue. Causes of depression mentioned include reproductive issues.	Many participants talked about dealing with their depression alone and using “inner strength.” The role of the GP was seen to be related to physical rather than mental illness.
(Kumari, 2004)	UK Community	First- and second-generation women from Indian sub-continent (Pakistan, Bangladesh, and India) N = 10 Mean = 33 Range = 16–60	Quantitative Questionnaire	Participants reported the following symptoms: over-feeling, tiredness, feeling low, and tension. 32% of the participants connected their aches and pains to unhappiness.	Help-seeking strategies included learning to deal with it or speaking to someone. Only 2/88 mentioned wanting to see a doctor. Other coping strategies cited were crying, relaxation and medication.
(Bhui et al., 2006)	UK Primary care, community	Bangladeshi, Black Caribbean, and White British individuals with mental distress N = 79 Mean = 40 Range = 19–77	Quantitative Checklist and interviews	The Bangladeshi participants were more likely to have a CMD and to refer to it as a physical illness than the Black Caribbean group. They were also more likely to give physical and spiritual explanations for the onset of CMDs, rather than behavioural issues. They saw the consequences of CMDs as psychological (96.2%) and social (49.4%).	The Bangladeshi participants found self-management (59.5%), social treatment (55.7%), and medical treatment (41.8%) the most helpful.
(Lavendera et al., 2006)	UK Primary care	Yoruba, Bangladeshi, and White British individuals N = 20 Range = 18–80 **Range not specific to South Asian participants	Qualitative Interviews	There was no consensus among the participants on whether depression could be categorised as an illness. Causes of depression noted were mainly attributed to family issues.	Most participants used friends and family as primary source of support. There were conflicting findings regarding the use of doctors and medicine.
(Lawrence et al., 2006a, 2006b)	UK Primary care	Black Caribbean, South Asian, and White British older adults N = 33 Range = 65–90	Qualitative Interviews	The South Asian participants in the “not depressed” group were more likely to see depression as an issue of personality (such as being weak or too sensitive) rather than a biological issue. Other causes of depression were family issues and grief. A consequence of depression was being unable to be around people and complete daily tasks. Participants were not likely to consider depression to be an illness.	Various strategies to cope with depression emerged such as self-help (participants thought that depression should be dealt with alone using cognitive techniques such as distraction and altering your attitude), social support (such as receiving support from family and/or friends), religion (such as meditating and visiting the temple), and finally formal healthcare (there was no consensus on the use of GPs for treating depression: some saw the value of taking medication, while others were concerned about the side effects).
(Hanley, 2007)	UK Community	Bangladeshi mothers with postnatal depression N = 10 Range = 16–24	Qualitative Focus group (N = 1)	Most of the participants did not consider postnatal depression to be an illness. Religious causes were often cited. Postnatal depression was described as “weakness, pain, problems of the heart.”	Primary sources of support included parents and the wider community. Results regarding views/use of healthcare professionals were mixed. Issues with disclosing to healthcare professionals included presence of husbands, shame about talking about pregnancy, and preferring to see religious leaders to cope. Other participants saw doctors and took prescribed medication.
(Mallinson & Popay, 2007)	UK Primary care	Individuals of Pakistani origin and White origin with depressive disorder N = 31 Range = 19–65	Qualitative Interviews	The participants were aware of the concept of depression and used this term. Depression was experienced as worrying, agitation, weakness, and feeling low and down. While the results on somatisation were unclear, other physical symptoms such as chills, feeling hot, and having a heavy and tense head were mentioned.	
(Rüdell et al., 2008)	UK Primary care, community	Bangladeshi, Black Caribbean, and White British N = 57 Mean =43	Mixed methods Survey		The Bangladeshi participants had many different help-seeking strategies. The group had the highest proportion of individuals who sought help from a GP. This group was also more likely to receive medication and find it helpful. 68.4% used social help-seeking (such as talking to family), 54.4% self-directed (such as exercise), 61.4% keeping busy, 28.1% seeing a traditional healer, 49.1% praying, 52.6% talking to GP, and 59.6% taking medication. This group preferred complementary strategies (such as massage and yoga) the least.
(Gask et al., 2011)	UK Primary care	British Pakistani women being treated for depression N = 15 Range = 23–73	Qualitative Interviews	Three primary themes that emerged from this study were: feeling stuck (unable to manage depression due to their relation to situational factors such as family conflict), isolation (due to stigma or self-imposed), and losing a sense of control (as a main experience of depression).	
(Rafique, 2010)	UK Specialist services	Pakistani women attending a counselling service for South Asian women N = 7 Mean = 36 Range = 24–48	Qualitative Interviews	Causes of depression were loneliness, life issues (such as family conflict), and physical illness. Participants reported physical symptoms such as not wanting to eat, fatigue, aching bodies, and psychological symptoms such as many emotions, inability to concentrate, memory issues, nonstop crying, and loneliness / self-imposed isolation.	Many of the participants did not want to discuss their issues with other people (i.e., not wanting to burden family) but were sometimes willing to speak to a professional. Coping strategies included keeping busy, seeing friends, reading, and using religion.
(Loewenthal et al., 2012)	UK Community, NHS Trust	Bengali, Urdu, Tamil, and Somali speaking individuals N = 71 All participants over 40	Qualitative Focus groups (N = 2)		Four primary themes that emerged from this study were: understanding of mental health issues and available services (lack of understanding of Western conceptualisations of depression), cultural barriers to approaching service providers (participants mentioned feeling isolated and coping alone), interpreter and GP services (results were mixed on the usefulness of GPs when handling depression or anxiety: issues of privacy and language barriers were cited), and religion (religious explanations for the occurrence of depression emerged, as did religious forms of coping, such as praying).
(Wittkowski et al., 2012)	UK Healthcare services (health visitors and midwives)	South Asian mothers N = 10	Qualitative Interviews	Causes of postnatal depression were described and included unhappy marriages, missing family in country of origin, or evil whispers. It was experienced as tension or constant overthinking. Western conceptualisations of depression were not used by this group.	The mothers were isolated and wanted support, but felt they could not get it from parents, husbands, or GPs due to cultural differences. Coping strategies included using religion, taking time for themselves, being optimistic, and trying to use family.
(Ekanayake et al., 2012)	Canada Community	Women of South Asian origin with symptoms of depression N = 10 Range = 22–65	Qualitative Interviews	Causes of depression cited were individual issues, family issues (cultural differences, divorce, bereavement), isolating, ageing, as well as migration and socioeconomic challenges.	
(Taylor et al., 2013)	UK Community	White British and North Indian women N = 70 Mean (SD) = 33 (10.6)	Quantitative Survey	When discussing a vignette of a mother with depression, the Indian group was less likely to feel like they understood her problems and that treatment would be helpful. They saw the cause as relationship or interpersonal challenges.	The Indian participants were also significantly less likely to believe the mother with depression should visit a GP. They endorsed seeing a religious or traditional healer. They also thought friends and family were a good source of support (at the same level as the White British group).
(McClelland et al., 2014)	UK Community	British Bangladeshi and British White individuals N = 190 Mean = 28 Range = 17–58	Quantitative Questionnaire	The Bangladeshi group was more likely than other groups to think shame is associated with depression and it can have an impact on the family of an individual with depression. They gave non-biological explanations of depression but did not endorse environmental explanations.	For treatment, the participants preferred relying on friends, family, religion, and self-coping than on medical intervention. The older Bangladeshi group was more likely to see depression as an illness versus the younger Bangladeshi group.
(Roberts et al., 2015)	USA Community	Asian Indian women N = 408 Mean (SD) Quantitative = 42 (15.38) Mean (SD) Qualitative unstated	Mixed methods Interviews (N = 11), focus groups (N = 4), survey (N = 350)	This study found differences between older- and younger- generation participants. One participant described how cultural differences between themselves and their parents were a cause for depression.	Stigma and fear of gossip in the community impeded help-seeking. The women participants were more likely to seek informal sources of support than professional mental health care. Help-seeking strategies differed between women who spoke Punjabi vs. English.
(Fernández de la Cruz et al., 2016)	UK Community	Ethnic minorities (including Indian parents) N = 47 Mean(SD) = 37(6.5) Range = 23–58	Quantitative Survey	Participants in this study were less likely to believe that OCD could lead to symptoms and that treatment would be helpful. They cited causes of their child's OCD as child's friends (95.5%), family or parenting issues (88.6%), or personality or emotional struggles (86.0%).	Indian parents possessed less information about OCD than White British parents. Indian parents mentioned they would seek help from a GP, family therapy, and family/friends.
(Antoniades et al., 2017, 2018)	Australia Community	Sri Lankan migrants and Anglo-Australians with depression N = 18 Mean = 41	Qualitative Interviews	The participants described feelings of hopelessness, agitation, nervousness, and entrapment. Somatisation did not emerge as an important theme. The depression was seen as a result of situational factors such as family conflict or attitudinal factors such as overthinking. Biological factors and religious factors were rarely mentioned.	Some participants saw depression as a temporary state and related this to not wanting to take medication. Other coping strategies included seeing a priest, distraction, and sitting and thinking. 84% of participants engaged with healthcare providers. Issues with parents emerged – some participants cited that their parents did not understand their depression, while others mentioned they required permission from their parents to receive mental health care. Participants also mentioned that they used “compatriots” as someone to talk to because they did not feel like their social network understood.
(Brijnath & Antoniades, 2018)	Australia Community	Indian Australians and Anglo-Australians N = 28 Mean (SD) = 40 (15.8)	Qualitative Interviews	Many participants had not disclosed their depression to friends/family due to stigma. They saw the consequence of their mental health issues as impacting their social lives, rather than work lives (in contrast to the Anglo-Australian participants).	They cited stigma in the wider community as being apparent and a determinant of help-seeking. However, there was less stigma regarding counselling in the Indian-Australian group than the Anglo-Australian group.
(Chiu et al., 2018)	Canada Community	White, South Asian (Indian, Pakistani, Bangladeshi, Sri-Lankan origin), Chinese, and Black residents N = 6779 Mean = 38	Quantitative Survey		Mental health service use was found to be lower among South Asian participants, though 51.4% of South Asians reported seeking help in the past year. The participants sought help from GPs more than other health professionals and were more likely to see their family doctors than other professionals.
(Gilbert et al., 2019)	Australia Community	Indian-Australians and Anglo Australians N = 36 *Mean = 53	Qualitative Focus groups (N = 10)	Four major findings emerged from this study for the Indian-Australian participants: 1) the nature of depression (depression as stemming from situation and attitudinal factors), 2) causes of depression (such as missing friends/family in country of origin, participants did not always see biomedical causes as legitimate)	3) Help-seeking (strategies such as prayer, meditation, attempting to forget, drinking tea emerged), and 4) moral legitimacy (participants saw depression as stemming from weakness/personality and it meant that it must be dealt with internally, rather than seeking help from strangers).
(Kateri et al., 2019)	Greece Community	First-generation Indian immigrants N = 114 Mean (SD)= 33 (7.97)	Quantitative Questionnaire	This study found that acculturation attitudes (‘separation’ where individuals prefer to maintain their culture of origin vs. ‘integration’) play an important role in how anxiety and depression is experienced.	
(Markova et al., 2020a, 2020b)	Norway Online	Immigrant groups (including Pakistani group) and Norwegian students N = 117 Mean (SD) = 29 (10.2)	Quantitative Questionnaire		The Pakistani participants preferred informal and traditional help-seeking more than the other ethnic groups. This was found to be related to maintenance of cultural orientation. The participants had the second highest preference for spiritual coping (after the Somali group) and endorsed other coping strategies such as exercise, time outside, reflection time, and looking for relationships. They used pluralistic help-seeking strategies.
(Birtel & Mitchell, 2023)	UK Online	White British and South Asian N = 46 Mean (SD) = 29 (7.62)	Quantitative Survey	The participants attributed depression to character flaws, God, and life circumstances, rather than biological reasons. The participants reported wanting more social distance and less closeness to a person with depression. They were also more likely than the White participants to report negative stigma by association.	

*Note*. The presented data is only for the relevant South Asian population. However, in one case, the age range is presented for the entire sample because the study did not disaggregate the data (**). Also, in some cases the mean age was calculated by the authors of this review (*).

In Table 1, we present the findings of the included studies in two categories (explanatory models and help-seeking), the definitions of which can be found in the eligibility criteria section of the Methods.

### Characteristics of included reports

Most of the reports were qualitative (n = 15), focusing exclusively on depressive disorders (n = 21), and explored a combination of explanatory models and help-seeking (n = 27). The studies were primarily based in the UK (n = 20). Other countries included Australia (n = 3), Canada (n = 2), Norway (n = 1), the United States (n = 2), and Greece (n = 1). The participants in the included reports were recruited online (n = 3), from the community (including educational institutes) (n = 16), healthcare settings (n = 11), or a combination of community and healthcare settings (n = 3). The ages of the participants ranged from 16 to 90. The year of publication of the included reports ranged from 1994 to 2022.

### Quality of included reports

The detailed quality assessment is summarised in the Supplementary Materials. The quality of the qualitative articles was generally high. The primary concern for seven out of 15 of the qualitative articles was inadequate consideration of the researcher–participant relationship. Another issue with some of the qualitative studies was insufficient detail on the data analysis process. For mixed methods articles, the authors justified why this study design was used, and both the qualitative and quantitative components were explained in detail. The quantitative articles were assessed to be of good quality. However, many samples were sourced from primary care services, and therefore over-represented individuals who engage with mainstream healthcare, with consequent implications for findings on help-seeking.

### Key themes: Explanatory models

#### Understandings of CMDs

*Descriptions of mental health:* Descriptions of mental health varied among the South Asian participants in the included studies. A 2011 UK-based study found that South Asian participants did not identify with diagnostic words based on Western understandings of CMDs. One Tamil participant drew on their experience working at a GP practice, saying, “Lots of people who come to us do not have a clue of what they are going through … there's still lack of knowledge and awareness among this community.” Another participant mentioned that there is no direct translation for “anxiety” in Urdu (Loewenthal et al., 2012). The participants in this study were more familiar with general mental health concepts and this topic sparked discussion on the term “pagol” (mad or crazy in Hindi). Conversely, in Mallin and Popay's study with 31 individuals of Pakistani origin (19 first-generation immigrants to the UK, 12 UK-born) recruited from a GP practice, it was found that participants were willing to use diagnostic terms such as depression (Mallinson & Popay, 2007). Those Pakistani-origin participants who did not use diagnostic terms such as depression or anxiety instead used descriptions such as “worried and on edge,” “really down and weak,” and “very low” (Mallinson & Popay, 2007, p. 864). In the 2017 Antoniades et al. study, it was found that Sri Lankan participants with depression used terms echoing “a sense of entrapment” (i.e., hopelessness), while the Anglo-Australian participants used terms to convey a “force weighing or dragging them down” (Antoniades et al., 2017, p. 5).

*Psychosocial definitions:* South Asian participants generally define depression in psychosocial (i.e., life circumstances) rather than biological (i.e., genetic factors or neurotransmitters) terms. For example, Commander et al. (2004) and Bhui et al.'s (2006) studies respectively found that 67% and 86.1% saw their CMD as a psychosocial issue. In McClelland et al.'s study, the British White participants were more likely to endorse biological explanations of depression, while the British Bangladeshi participants were more likely to endorse non-biological explanations such as a boring life (McClelland et al., 2014).

#### Perceived causes of CMDs

*Situational and moral factors:* Many South Asian participants in the included studies saw the cause of their CMD as arising from situational factors, rather than biological factors (Roberts et al., 2015; Taylor et al., 2013). They mentioned family conflict, grief/bereavement, missing their family in their country of origin, and generation gaps with their children as possible causes. One participant in Gask et al.'s (2011) study said that their child was disrespectful to their elders and this “comes in form of depression for parents” (p. 53). In a study with South Asian parents of children with obsessive compulsive disorder, participants cited children's friends and parenting/family issues as the most likely causes (Fernández de la Cruz et al., 2016). This was also mirrored in Birtel and Mitchell’s (2023) study where South Asian participants were found to “endorse greater supernatural but also moral beliefs [such as character flaws] about the causes of depression than White British” (p. 11).

*Spiritual/religious factors:* Participants gave spiritual/religious explanations for CMDs (Hanley, 2007), though less frequently than anticipated. In some studies, participants saw their distress because of “Jinn,” a supernatural being in Islam, or “evil whisperings” (Lavendera et al., 2006; Loewenthal et al., 2012; Wittkowski et al., 2012). In Hanley's (2007) study of South Asian mothers with post-natal depression, religious causes were often cited. However, in a qualitative study with South Asian women with depression in Toronto, no spiritual explanations were given (Ekanayake et al., 2012) and in a study of Sri Lankan Australians with depression, only one spiritual explanation was given (Antoniades et al., 2018).

#### Experiences of CMDs

*Symptoms:* Common symptoms expressed were crying, tiredness, hopelessness, aches and pains, isolation (Bhui et al., 2001; Kumari, 2004), “thinking too much,” having “too many emotions” (Rafique, 2010; Wittkowski et al., 2012), trouble concentrating, memory loss, mental tension, and pressure/pain in the head (Lawrence et al., 2006a, 2006b; Rafique, 2010). In Bhui et al.'s (2002) study, it was found that Punjabi individuals with CMD were more likely to report poor concentration and memory than English individuals. Punjabi individuals were also more likely to express their CMD as somatic symptoms than English individuals.

*Consequences of CMDs:* CMDs were conceptualised as stigmatised issues with social consequences. A 25-year-old male Indian-Australian participant reported, “It's a social statement that, ‘Oh he has depression, I don’t want to get close to him.’ And it's almost like people think it's contagious” (Brijnath & Antoniades, 2018, p. 8). The same study showed that South Asian Australians were more likely to focus on the social impact of their depression, in contrast to Anglo-Australians who were more concerned with the work impact (Brijnath & Antoniades, 2018). Additionally, a mixed methods study in California found that fear of gossip in the South Asian community was directly related to anxiety (Roberts et al., 2015).

#### Generational differences in explanatory models of CMDs

Cultural differences in the understanding and experience of CMDs between parents and their children or older and younger generations emerged as a common theme on CMD cause, experience, and help-seeking.

In some studies, participants noted that their CMD stemmed from cultural differences (e.g., thoughts on marriage, rules, Westernisation) between themselves and their parents and/or other family members (Ekanayake et al., 2012). In Roberts et al.'s (2015, p. 11) study with Punjabi individuals in California, one participant said “… as an adolescent it's depressing … you’re growing up in this first-generation culture where it's like your parents don’t understand where you’re coming from … there can be times that it can be, you know, really depressing.” Additionally, Farver et al.'s (2002) study found that adolescents were more likely to be assimilated to their country of residence than their parents and that families with larger acculturation gaps had higher anxiety scores.

Beyond cultural differences being a root of CMD development, disparities in the understanding of, and attitudes towards, CMDs between parents and their children and/or older and younger generations were also apparent. In McClelland et al.'s (2014) study, the older British Bangladeshi participants were more likely to demonstrate negative perceptions of depression younger British Bangladeshis and British Whites. The older British Bangladeshi participants also had more stigmatising beliefs about depression like it resulting from personal failures and leading to consequences for the family's respect (McClelland et al., 2014). Similarly, Furnham and Malik (1994) found that the young South Asian sample in their study held beliefs on depression that were consistent with their British counterparts. Conversely, for example, an older-generation South Asian participant stated that “the depression that exists in Indian-Australians, it really is nothing, it's just the style of living” (Gilbert et al., 2019, p. 297). This result was mirrored in Antoniades et al.'s (2018) study. A 22-year-old participant stated: “my parents were like ‘there is no such thing as depression … no one is depressed in Sri Lanka” (p. 6).

### Key themes: Help-seeking

#### Informal care

Many self-management techniques for depression and anxiety were reported, such as distraction (“keeping busy”), getting married, crying, exercise, altering your perspective to a more positive one, and “sitting and thinking” (Brijnath & Antoniades, 2018; Gask et al., 2011; Kumari, 2004; Markova et al., 2020a, 2020b; Rüdell et al., 2008). In Norway, Markova et al. (2020a, 2020b) found that Pakistani immigrants preferred disengagement coping strategies (such as avoidance, distraction, and finding a partner) in comparison to other immigrant groups and Norwegian students (Markova et al., 2020a, 2020b). While some participants reported wanting to keep their mental health issues private, others described talking to family and friends as a source of support. A mixed methods study with Bangladeshi individuals in the UK found that 68.4% used social help-seeking (i.e., speaking with your family) (Rüdell et al., 2008). Additionally, spiritual coping strategies such as praying or visiting a faith healer were frequently cited in studies examining help-seeking (Taylor et al., 2013). Finally, one study highlighted how participants sought support from “compatriots” online, who in this case were people with lived experiences or those who had a deep understanding of mental health issues (Antoniades et al., 2018).

#### Formal care

South Asian participants’ views on attending primary care to receive treatment for depression and/or anxiety were mixed. Some reported not wanting to see a GP for fear of judgement, issues of confidentiality, miscommunication, only going to the GP for physical health problems, and lack of access (Gask et al., 2011). For example, Wittkowski et al.'s (2012) study with 10 South Asian mothers with postnatal depression highlighted that they did not feel comfortable going to a male GP, especially since they thought they would be judged for the number of children they had. In Kumari's (2004) qualitative survey, only 2/88 participants (who were all in counselling) said they would see a doctor for mental health problems. Similarly, a participant in another study mentioned that depression is a problem of the mind and as such “doctors’ medicine cannot work” (Lavender et al., 2006, p. 655) Conversely, participants in another study stated that “the doctor should decide what is best for the patient as he knows better” (Gask et al., 2011, p. 52). Another participant believed that medication could positively impact the mind (Lavendera et al., 2006). In Rüdell et al.'s (2008) study, Bangladeshi participants were more likely than their White British and Black Caribbean peers to visit a GP, receive medication, and find it helpful.

A Canadian survey found that South Asians underutilised mental health care services; 51.4% of South Asians with suicidal ideation reported seeking help for their mental health concerns in the past year (Chiu et al., 2018). In another study, it was found that most South Asian participants had contacted a family doctor but only 55% had discussed their mental health concerns (Commander et al., 2004). In Mallinson and Popay's (2007) quantitative study, White women and Pakistani individuals with depression were found to visit GPs a similar number of times for symptoms of depression of anxiety, “GP consultation rates were higher in depressed people of Pakistani origin because they consulted more often for bodily symptoms” (p. 862).

Finally, perspectives on strategies to address cultural issues in primary care were highlighted. A study based in a community health centre for South Asian women found that participants wanted to attend healthcare services with professionals of their own cultural background (Kumari, 2004). The presence of interpreters at GP services was not found to be useful to some because of mistranslation issues and preferring to speak to their doctor directly and confidentially (Loewenthal et al., 2012). Some participants in Lavendera et al.’s (2006) study recommended the use of mullahs (Islamic religious leaders) alongside GPs: “Without mullah, doctor cannot do anything.”

#### Generational differences in help-seeking

Furnham and Malik’s (1994) study found that younger-generation participants preferred speaking to friends for support, while the older generation preferred to speak with family. One explanation some participants cited was that their family members lacked understanding of mental health and would therefore be unable to help: “they just have absolutely no understanding of it … if I said to my mum, all she is going to do is pray” (Antoniades et al., 2018, p. 8). Other participants mentioned that they would not seek help from mental health professionals without permission from their parents (Antoniades et al., 2018): “I would get permission from my parents first and then I would go there” (p. 6). Antoniades et al.'s study also illustrated the role that family plays in promoting help-seeking behaviour. The Anglo-Australian participants were more likely to be encouraged by their friends, while for Sri Lankan Australians family members were more important (Antoniades et al., 2018).

## Discussion

To our knowledge, this is the first systematic review that aimed to synthesise the evidence on explanatory models of CMDs and help-seeking among South Asians living in HICs. The following sections highlight our key findings.

### Explanatory models

The South Asian participants in the included studies primarily described CMDs in non-diagnostic terms such as “feeling low,” although some participants were comfortable using labels such as “depression” or “anxiety.” These descriptions were echoed in Awan et al.'s (2022) systematic review; South Asian participants with long-term conditions were more likely to use words such as “tension,” “stress,” or “anger” to explain their emotional distress than medical terms. This use of non-diagnostic terminology is likely related to how South Asian individuals often frame CMDs as psychosocial (resulting from factors such as family conflict or personality traits) or religious issues rather than biological. It may therefore be necessary to invest in the development of culturally adapted psychosocial interventions for this population in addition to pharmacological interventions. For example, psychosocial group interventions (“structured cognitive, behavioural and social interventions intended to improve mental health implemented among a group of people who meet together on multiple occasions” (Mathias et al., 2023, p. 2)) run in South Asia were shown to improve mental health outcomes at intrapersonal, interpersonal, and community levels because they increased participants’ awareness of mental health tools and resources, provided trusting relationships, and increased their feelings of social support and inclusion (Mathias et al., 2023).

Stigma also influences the explanatory models of CMDs for this population. The included studies showed that many South Asian participants were concerned about the social consequences of their CMD, such as being labelled as “weak” or “crazy” in their family or wider community. Based on the definitions of Corrigan and Rao (2012), two types of stigma are particularly important for the South Asian diaspora in HICs; public stigma (“the prejudice and discrimination directed at a group by the larger population”) and self-stigma (“when people internalize these public attitudes and suffer numerous negative consequences as a result”) (Corrigan & Rao, 2012). Addressing all forms of stigma in South Asian communities is crucial to increase help-seeking and improve mental health outcomes. In Naeem et al.'s (2020) “call to action,” the authors bring attention to how stigma acts as a barrier to accessing support for South Asian Canadians. When discussing strategies to address stigma, they point to Fung et al.'s (2022) study with 495 Asian men in Toronto (including South Asian men). Stigma-reducing interventions such as acceptance and commitment therapy (ACT), contact-based empowerment education, or both, and psychoeducation were found to be effective due to mediating factors such as empowerment and, for ACT, “psychological flexibility.”

### Family dynamics

Family dynamics played an important role in how South Asian individuals perceived the cause, impact, and experience of their CMD, which in turn influenced their preferred coping strategies. For some participants, family acted as a protective factor and their primary support, while for others family acted as a cause for CMD and a barrier to help-seeking. These mixed results are echoed in Anand and Cochrane’s (2005) review of British South Asians’ mental health status, where they found that for this group “family is both a source of strength and a source of stress.”

Family issues, a psychosocial factor, were cited as a cause for the onset of depression or anxiety in multiple studies (Antoniades et al., 2017; Ekanayake et al., 2012; Rafique, 2010; Wittkowski et al., 2012) . Our results highlighted that one of the most significant issues described was conflict arising from cultural differences within families, especially between first-generation parents and their second-generation children or grandchildren. Similarly, in a recent study, it was found that grandmothers with granddaughters who considered themselves “Hindu” or “Indian” over British, had better psychological adjustment scores (Guglani et al., 2000). Second-generation children, meanwhile, have the pressure of balancing their family culture and their host country's culture, which can lead to heightened levels of stress and anxiety (Shariff, 2009). These findings also appear to be true from the perspective of healthcare providers. Rastogi et al. (2014) and Islam et al. (2022) interviewed clinicians and mental health workers with experience of working with people of South Asian origin in the United States and Canada respectively and both groups of study participants perceived cultural differences (in ideas about mental health, academics, dating, and clothing, for example) to be a cause for the onset of CMDs and a barrier to mental healthcare (Islam et al., 2022; Rastogi et al., 2014). Also, while this review was specific to HICs, attributing a CMD to life circumstances has also been seen in low-income settings. For example, a study of primary care attenders with a CMD in India found that the most common explanation for their illness was psychosocial factors such as marital strife and concerns about family members with alcoholism (Patel et al., 1998).

We also found that many participants were primarily concerned with the impact their CMD would have on their family, which is unsurprising given that it is well established that South Asians endorse a collectivist orientation where the family is prioritised over the individual. In some cases, it emerged that CMDs were not discussed with family in order to protect them from pain, worry, or humiliation (Brijnath & Antoniades, 2018). This finding was echoed in Rafique’s (2010) study, where participants did not want to concern their family with their worries, and in Loewenthal et al.'s (2012) study where an older Tamil participant stated that they turned to religion as a coping strategy to remain “strong” for their family.

Conversely, other participants stated that support from family was essential to combating depression and that they would rather share their mental health issues with family than with a professional (Hanley, 2007; Lawrence et al., 2006a, 2006b). This finding is supported by the theory that perceived support from a spouse or family can reduce depressive symptoms (Singla et al., 2021). Further, family played an important role in accessing primary care services, which ranged from wanting a husband to be present when seeing a male doctor (Wittkowski et al., 2012) to requiring their child to be present to translate from their spoken language to English (Loewenthal et al., 2012). These results were also mirrored in Prajabati and Liebling's (2022) systematic review of South Asians and mental health service use in the UK, which found that “family was viewed as the main source of support.”

### Coping strategies

Individual coping strategies such as crying, praying, exercise, isolation, or thinking were a prominent finding in this review. One proposed reason is that the familial factors often translate to private coping strategies as they avoid issues of shame, stigma, and familial burden (Cinnirella & Loewenthal, 1999). This may be especially true for girls and women. For example, in Loewenthal et al.'s (2012) study, in addition to stigma, the female participants expressed that they wanted to remain strong (and therefore not seek help) given the patriarchal nature of the community (Loewenthal et al., 2012). This finding is mirrored in a 2021 study with British-born South Asian girls, which found that the “desire to avoid disrupting cultural norms can close down opportunities to seek help” (Sangar & Howe, 2021, p. 19). Additionally, a study in Scotland found that shame resulting from stigmatised views on mental health problems decreased social and professional help-seeking in Indian, Pakistani, and Chinese communities in Scotland (Knifton, 2012).

Besides family considerations, there are other plausible explanations for why self-reliance and individual coping strategies (such as using “inner strength”) were found to be popular in the included studies. We found that South Asian individuals attributed depression/anxiety to attitudinal factors (Lawrence et al., 2006a, 2006b). This was also seen in a study that found that South Asian students were more likely to link character deficit to mental illness than their White counterparts (Mokkarala et al., 2016). Considering that “cultural causal beliefs about mental distress [are] significant predictors of attitudes for seeking help” (Sheikh & Furnham, 2000, p. 326), we can posit that viewing CMDs as a consequence of personal problems is correlated to the use of individual help-seeking strategies.

### Implications for clinicians and service delivery

To better integrate culture into mental health service delivery in high-income settings, the following should be considered. First, it is important for healthcare professionals to acknowledge, and therefore make time for, exploring their South Asian patients’ cultural context in order to deliver person-centred care (Keynejad, 2011). Efforts in clinical settings have escalated in recent years through the implementation of tools such as the Cultural Formulation Interview, which is a guide to help clinicians elicit information on their patients’ social and cultural context. However, there are some limitations. In a recent review of its trial, Jarvis et al. (2020) highlighted that some clinicians without social sciences backgrounds struggled to understand a question intending to determine “clinically relevant aspects of the patient's cultural identity” (p. 41). Second, healthcare professionals and decision-makers must be aware of the significant role that families and differing immigrant generation status play in the development and treatment of, and recovery from, CMDs. In the Kingdom of Saudia Arabia, for example, pharmacological intervention (i.e., prescribing medication) was the most common form of support offered to patients for mental health issues (71%), compared to family therapy (8%) (Algahtani et al., 2017; Rathod et al., 2017). A study with South Asian families in the United States has suggested that care providers allow for differences in values between parents and their children when addressing mental health concerns (Sharma et al., 2020). Culturally adapted family therapy could use the tool of “cultural brokering” in which issues between first-generation South Asian parents and their second-generation children are reframed as a difference in cultural values, rather than a problem stemming from an individual (Segal, 2018; Shariff, 2009). Third, strengthening and diversifying the healthcare workforce may make care more accessible to some South Asian individuals, which can include involving traditional or faith healers in primary care or working with key community members to deliver educational workshops (Keynejad, 2011; Shah et al., 2023). Fourth, community-based interventions, outside of the traditional healthcare system, need to be considered to increase access to care for South Asians with CMD. Digital technology could offer an important avenue to deliver said interventions: “these platforms can be designed with features that are culturally tailored and available in multiple languages” (Shah et al., 2023, p. 382). Finally, future research should focus on South Asian sub-groups (i.e., by country of origin) and immigration generation to develop prevention strategies and service delivery (Prajabati & Liebling, 2022).

### Limitations

This review had some limitations. First, we only included English publications. However, considering that most research from HICs is published in English journals, it is unlikely that we have missed publications that are published in non-English languages. Second, some articles in the search did not define South Asian in their study context and were excluded. Therefore, some articles may have met this review's inclusion criteria but were excluded due to lack of detailed definitions. Third, most of the studies were based in the UK, which potentially limits the generalisability of these findings, considering that a host country's political-social-cultural environment can impact an individual's explanatory models. Fourth, inter-rater reliability was not calculated between the reviewers for the data selection process, which could impact the reliability of the review. Fifth, considering that this is an exploratory systematic review, we did not weight the findings of the included reports based on the quality assessment.

Importantly, the selected articles included varying sub-groups within the group of “South Asian,” which meant that sub-group analysis by country of origin was not possible. This limitation is important given the economic, political, cultural, and social differences that exist within this group (Prajapati & Liebling, 2022). Additionally, many articles were not clear about the immigration generation of the South Asian participants, which is important considering that the characteristics associated with ethnicity, passed through generations, could shift through processes such as migration (Durà-Vilà & Hodes, 2012; Rutter & Tienda, 2005). Nevertheless, it was possible to make some conclusions on how the views of parents and their children converge and/or differ regarding CMD understanding, experience, and help-seeking.

## Conclusion

Academics, policy makers, and clinicians must work with South Asian communities in their local context to achieve “vertical equity” where healthcare services are tailored to meet the needs and expectations of diverse groups, as opposed to “horizontal equity” where identical services exist for everyone (Tribe & Marshall, 2020). In the past, even when efforts have been made to address issues of diversity and achieve equity in the healthcare systems of HICs, there are “numerous examples of stereotyping of specific cultural groups leading to interventions that are often inadequate or inappropriate” (Gopalkrishnan, 2018, p. 3). An important step in achieving vertical equity is to continuously develop the evidence base on how culture influences explanatory models of, and help-seeking for, CMDs (Deacon, 2013). Exploring the cultural influences on the explanatory models of, and help-seeking for, mental healthcare among South Asian individuals, which this review aimed to do, can provide valuable knowledge to mental health service providers who work in diverse contexts.

## Supplemental Material

sj-docx-1-tps-10.1177_13634615241296302 - Supplemental material for Explanatory models of common mental disorders among South Asians in high-income countries: A systematic review Supplemental material, sj-docx-1-tps-10.1177_13634615241296302 for Explanatory models of common mental disorders among South Asians in high-income countries: A systematic review by Ruchika Jain, Ritsuko Kakuma, Daisy R. Singla, Kirsty Andresen, Khawater Bahkali, and Abhijit Nadkarni in Transcultural Psychiatry

## References

[bibr1-13634615241296302] Ahmad G. McManus S. Cooper C. Hatch S. L. Das-Munshi J. (2021). Prevalence of common mental disorders and treatment receipt for people from ethnic minority backgrounds in England: Repeated cross-sectional surveys of the general population in 2007 and 2014. The British Journal of Psychiatry, 221(3), 520–527. 10.1192/BJP.2021.179 PMC7616892 35049474

[bibr2-13634615241296302] Algahtani H. Buraik Y. Ad-Dab’bagh Y. (2017). Psychotherapy in Saudi Arabia: Its history and cultural context. Journal of Contemporary Psychotherapy, 47(2), 105–117. 10.1007/S10879-016-9347-2/METRICS

[bibr3-13634615241296302] Anand A. S. Cochrane R. (2005). The mental health status of south Asian women in Britain: A review of the UK literature. Psychology and Developing Societies, 17(2), 195–214. 10.1177/097133360501700207

[bibr4-13634615241296302] Antoniades J. Mazza D. Brijnath B. (2017). Becoming a patient-illness representations of depression of Anglo-Australian and Sri Lankan patients through the lens of Leventhal’s illness representational model. International Journal of Social Psychiatry, 63(7), 569–579. 10.1177/0020764017723669 28786331

[bibr5-13634615241296302] Antoniades J. Mazza D. Brijnath B. (2018). Agency, activation and compatriots: The influence of social networks on health-seeking behaviours among Sri Lankan migrants and Anglo-Australians with depression. Sociology of Health and Illness, 40(8), 1376–1390. 10.1111/1467-9566.12764 29998582

[bibr6-13634615241296302] Arnault D. S. (2009). Cultural determinants of help seeking: A model for research and practice. Research and Theory for Nursing Practice, 23(4), 259–278. 10.1891/1541-6577.23.4.259 19999745 PMC2796597

[bibr7-13634615241296302] Awan H. Mughal F. Kingstone T. Chew-Graham C. A. Corp N. (2022). Emotional distress, anxiety, and depression in South Asians with long-term conditions: A qualitative systematic review. *British Journal of General Practice* , *72*(716), e179–e189. https://doi.org/10.3399/bjgp.2021.0345 10.3399/bjgp.2021.0345 PMC8884439 35131838

[bibr8-13634615241296302] Beiser M. Simich L. Pandalangat N. (2003). Community in distress: Mental health needs and help-seeking in the Tamil community in Toronto. International Migration, 41(5), 233–245. 10.1111/J.0020-7985.2003.00268.X

[bibr9-13634615241296302] Bhavsar V. Ventriglio A. Bhugra D. (2021). The mental health of South Asians in the UK. In D. Moussaoui, D. Bhugra, & A. Ventriglio, (Eds), Mental health and illness in migration (pp. 1–133). Mental Health and Illness Worldwide.

[bibr10-13634615241296302] Bhugra D. (2004). Migration and mental health. Acta Psychiatrica Scandinavica, 109(4), 243–258. 10.1046/J.0001-690X.2003.00246.X 15008797

[bibr11-13634615241296302] Bhui K. Bhugra D. (2002). Mental illness in Black and Asian ethnic minorities: Pathways to care and outcomes. Advances in Psychiatric Treatment, 8(1), 26–33. 10.1192/APT.8.1.26

[bibr12-13634615241296302] Bhui K. Bhugra D. (2003). Explanatory models in psychiatry [1]. British Journal of Psychiatry, 183, 170. 10.1192/BJP.183.2.170 12893672

[bibr13-13634615241296302] Bhui K. Bhugra D. Goldberg D. (2002). Causal explanations of distress and general practitioners’ assessments of common mental disorder among Punjabi and English attendees. Social Psychiatry and Psychiatric Epidemiology, 37, 38–45. 10.1007/s127-002-8212-9 11924748

[bibr14-13634615241296302] Bhui K. Bhugra D. Goldberg D. Dunn G. Desai M. (2001). Cultural influences on the prevalence of common mental disorder, general practitioners’ assessments and help-seeking among Punjabi and English people visiting their general practitioner. Psychological Medicine, 31(5), 815–825. 10.1017/S0033291701003853 11459379

[bibr15-13634615241296302] Bhui K. McCabe R. Weich S. Singh S. Johnson M. Szczepura A. (2013). THERACOM: A systematic review of the evidence base for interventions to improve therapeutic communications between black and minority ethnic populations and staff in specialist mental health services. Systematic Reviews, 2, 15. 10.1186/2046-4053-2-15 23442299 PMC3599664

[bibr16-13634615241296302] Bhui K. Rüdell K. Priebe S. (2006). Assessing explanatory models for common mental disorders. Journal of Clinical Psychiatry, 67, 964–971. 10.4088/JCP.v67n0614 16848657

[bibr17-13634615241296302] Bhui K. Stansfeld S. Hull S. Priebe S. Mole F. Feder G. (2003). Ethnic variations in pathways to and use of specialist mental health services in the UK: Systematic review. British Journal of Psychiatry, 182, 105–116. 10.1192/BJP.182.2.105 12562737

[bibr18-13634615241296302] Birtel M. D. Mitchell B. L. (2023). Cross-cultural differences in depression between White British and South Asians: Causal attributions, stigma by association, discriminatory potential. Psychology and Psychotherapy: Theory, Research and Practice, 96(1), 101–116. 10.1111/papt.12428 PMC10092833 36300674

[bibr19-13634615241296302] Bismar D. (2018). Mental illness stigma, parent-child communication, and help seeking of young American adults with immigrant parents. Journal of College Counseling, 24(2), 146–161. 10.1002/jocc.12182

[bibr20-13634615241296302] Bowl R. (2007). The need for change in UK mental health services: South Asian service users’ views. Ethnicity and Health, 12(1), 1–19. 10.1080/13557850601002239 17132582

[bibr21-13634615241296302] Bradby H. Varyani M. Oglethorpe R. Raine W. White I. Helen M. (2007). British Asian families and the use of child and adolescent mental health services: A qualitative study of a hard to reach group. Social Science and Medicine, 65(12), 2413–2424. 10.1016/J.SOCSCIMED.2007.07.025 17766019

[bibr22-13634615241296302] Brijnath B. Antoniades J. (2018). What is at stake? Exploring the moral experience of stigma with Indian-Australians and Anglo-Australians living with depression. Transcultural Psychiatry, 55(2), 178–197. 10.1177/1363461518756519 29411686

[bibr23-13634615241296302] Burr J. Chapman T. (2004). Contextualising experiences of depression in women from South Asian communities: A discursive approach. Sociology of Health & Illness, 26(4) 433–452. 10.1111/j.0141-9889.2004.00398.x 15268700

[bibr24-13634615241296302] CASP. (n.d.). *CASP checklists – Critical appraisal skills programme*. https://casp-uk.net/casp-tools-checklists/

[bibr25-13634615241296302] Chiu M. Amartey A. Wang X. Kurdyak P. (2018). Ethnic differences in mental health status and service utilization: A population-based study in Ontario, Canada. Canadian Journal of Psychiatry, 63(7), 481–491. 10.1177/0706743717741061 29514512 PMC6099776

[bibr26-13634615241296302] Cinnirella M. Loewenthal K. M. (1999). Religious and ethnic group influences on beliefs about mental illness: A qualitative interview study. The British Journal of Medical Psychology, 72(4, Pt 4), 505–524. 10.1348/000711299160202 10616133

[bibr27-13634615241296302] Close C. Kouvonen A. Bosqui T. Patel K. O’Reilly D. Donnelly M. (2016). The mental health and wellbeing of first generation migrants: a systematic-narrative review of reviews. Globalization and Health, 12(1), 1–13. 10.1186/S12992-016-0187-3 PMC4997738 27558472

[bibr28-13634615241296302] Commander M. J. Odell S. M. Surtees P. G. Sashidharan S. P. (2004). Care pathways for South Asian and white people with depressive and anxiety disorders in the community. Social Psychiatry and Psychiatric Epidemiology, 39(4), 259–264. 10.1007/s00127-004-0736-6 15085326

[bibr29-13634615241296302] Corrigan P. W. Rao D. (2012). On the self-stigma of mental illness: Stages, disclosure, and strategies for change. Canadian Journal of Psychiatry. Revue Canadienne de Psychiatrie, 57(8), 464. 10.1177/070674371205700804 22854028 PMC3610943

[bibr30-13634615241296302] Crowley G. (2022). Comment on ‘Time for hard choices – a new global order for mental health’: Why migrants should no longer be ignored. International Journal of Social Psychiatry 69(1), 233–234. 10.1177/00207640221112322/FORMAT/EPUB PMC9936432 35979552

[bibr31-13634615241296302] Deacon B. J. (2013). The biomedical model of mental disorder: A critical analysis of its validity, utility, and effects on psychotherapy research. Clinical Psychology Review, 33(7), 846–861. 10.1016/j.cpr.2012.09.007 23664634

[bibr32-13634615241296302] Delara M. (2016). Social determinants of immigrant women’s mental health. Advances in Public Health, 2016, 1–11. 10.1155/2016/9730162

[bibr33-13634615241296302] Dinos S. Ascoli M. Owiti J. A. Bhui K. (2017). Assessing explanatory models and health beliefs: An essential but overlooked competency for clinicians. BJPsych Advances, 23(2), 106–114. 10.1192/APT.BP.114.013680

[bibr34-13634615241296302] Durà-Vilà G. Hodes M. (2012). Ethnic factors in mental health service utilisation among people with intellectual disability in high-income countries: Systematic review. Journal of Intellectual Disability Research, 56(9), 827–842. 10.1111/J.1365-2788.2011.01466.X 21883599

[bibr35-13634615241296302] Ekanayake S. Ahmad F. McKenzie K. (2012). Qualitative cross-sectional study of the perceived causes of depression in South Asian origin women in Toronto. BMJ Open, 2(1). 10.1136/bmjopen-2011-000641 PMC3282289 22337816

[bibr36-13634615241296302] Farver J. A. M. Narang S. K. Bhadha B. R. (2002). East meets west: Ethnic identity, acculturation, and conflict in Asian Indian families. Journal of Family Psychology, 16(3), 338–350. 10.1037/0893-3200.16.3.338 12238415

[bibr37-13634615241296302] Fernández de la Cruz L. Kolvenbach S. Vidal-Ribas P. Jassi A. Llorens M. Patel N. Weinman J. Hatch S. L. Bhugra D. Mataix-Cols D. (2016). Illness perception, help-seeking attitudes, and knowledge related to obsessive–compulsive disorder across different ethnic groups: A community survey. Social Psychiatry and Psychiatric Epidemiology, 51(3), 455–464. 10.1007/s00127-015-1144-9 26498926

[bibr38-13634615241296302] Fernando S. (2014). Globalization of psychiatry – A barrier to mental health development. *International Review of Psychiatry*, 26(5), 551–557. 10.3109/09540261.2014.920305 25343630

[bibr39-13634615241296302] Fung K. P. L. Liu J. J. W. Wong J. P. H. (2022). Exploring mechanisms of mental illness stigma reduction in Asian Canadian men. Canadian Journal of Psychiatry. Revue Canadienne de Psychiatrie, 67(6), 490. 10.1177/07067437211018674 34027706 PMC9149531

[bibr40-13634615241296302] Furnham A. Malik R. (1994). Cross-cultural beliefs about depression. International Journal of Social Psychiatry, 40(2), 126–133. 10.1177/002076409404000203 7989173

[bibr41-13634615241296302] Gadalla T. M. (2010). Ethnicity and seeking treatment for depression: A Canadian national study. Canadian Ethnic Studies, 41(3), 233–245. 10.1353/CES.2010.0042

[bibr42-13634615241296302] Gask L. Aseem S. Waquas A. Waheed W. (2011). Isolation, feeling “stuck” and loss of control: Understanding persistence of depression in British Pakistani women. Journal of Affective Disorders, 128(1–2), 49–55. 10.1016/j.jad.2010.06.023 20633932

[bibr43-13634615241296302] Gilbert A. S. Antoniades J. Bowen Z. Brijnath B. (2019). Legitimising depression: Community perspectives and the help-seeking continuum. Health Sociology Review, 28(3), 291–306. 10.1080/14461242.2019.1670090

[bibr44-13634615241296302] Gilbert P. Gilbert J. Sanghera J. (2006). A focus group exploration of the impact of izzat, shame, subordination and entrapment on mental health and service use in South Asian women living in Derby. Mental Health, Religion & Culture, 7(2), 109–130. 10.1080/13674670310001602418

[bibr45-13634615241296302] Gopalkrishnan N. (2018). Cultural diversity and mental health: Considerations for policy and practice. Frontiers in Public Health, 6, 179. 10.3389/FPUBH.2018.00179 29971226 PMC6018386

[bibr46-13634615241296302] Guglani S. Coleman P. G. Sonuga-Barke E. J. S. (2000). Mental health of elderly Asians in Britain: A comparison of Hindus from nuclear and extended families of differing cultural identities. International Journal of Geriatric Psychiatry, 15(11): 1046–1053. 11113985 10.1002/1099-1166(200011)15:11<1046::aid-gps229>3.0.co;2-c

[bibr47-13634615241296302] Gunasinghe C. Hatch S. L. Lawrence J. (2019). Young Muslim Pakistani women’s lived experiences of izzat, mental health, and well-being. Qualitative Health Research, 29(5), 747–757. 10.1177/1049732318803094 30293483

[bibr48-13634615241296302] Hanley J. (2007). The emotional wellbeing of Bangladeshi mothers during the postnatal period. Community Practitioner, 80(5), 34–37. 17536469

[bibr49-13634615241296302] Helman C. (1994). Culture, health, and illness: An introduction for health professionals. Butterworth-Heinemann.

[bibr50-13634615241296302] Hong Q. N. Pluye P. Fàbregues S. Bartlett G. Boardman F. Cargo M. Dagenais P. Gagnon M.-P. Griffiths F. Nicolau B. Rousseau M.-C. Vedel I. (n.d.). Mixed Methods Appraisal Tool (MMAT) version 2018 user guide. http://mixedmethodsappraisaltoolpublic.pbworks.com/ 10.1016/j.jclinepi.2019.03.008 30905698

[bibr51-13634615241296302] Hussain F. A. Cochrane R. (2010). Living with depression: Coping strategies used by South Asian women, living in the UK, suffering from depression. Mental Health, Religion & Culture, 6(1), 21–44. 10.1080/1367467021000014864

[bibr52-13634615241296302] Hussain F. Cochrane R. (2016). Depression in South Asian women living in the UK: A review of the literature with implications for service provision. Transcultural Psychiatry, 41(2), 253–270. 10.1177/1363461504043567 15446723

[bibr53-13634615241296302] Islam F. Khanlou N. Tamim H. (2014). South Asian populations in Canada: Migration and mental health. BMC Psychiatry, 14(1). 10.1186/1471-244X-14-154 PMC4052282 24884792

[bibr54-13634615241296302] Islam F. Qasim S. Ali M. Hynie M. Shakya Y. McKenzie K. (2022). South Asian youth mental health in Peel Region, Canada: Service provider perspectives. Transcultural Psychiatry, 60(2), 368–382. 10.1177/13634615221119384 PMC10164228 36113160

[bibr55-13634615241296302] Jacob K. S. Bhugra D. Lloyd K. R. Mann A. H. (1998). Common mental disorders, explanatory models and consultation behaviour among Indian women living in the UK. Journal of the Royal Society of Medicine, 91(2), 66–71. 10.1177/014107689809100204 9602740 PMC1296487

[bibr56-13634615241296302] Jarvis G. E. Kirmayer L. J. Gómez-Carrillo A. Aggarwal N. K. Lewis-Fernández R. (2020). Update on the cultural formulation interview. Psychiatry, 18(1), 40–46. 10.1176/APPI.FOCUS.20190037 PMC7011218 32047396

[bibr57-13634615241296302] Kapadia D. Nazroo J. Tranmer M. (2018). Ethnic differences in women’s use of mental health services: Do social networks play a role? Findings from a national survey. Ethnicity and Health, 23(3), 293–306. 10.1080/13557858.2016.1263283 27892690

[bibr58-13634615241296302] Karasz A. (2005). Cultural differences in conceptual models of depression. Social Science and Medicine, 60(7), 1625–1635. 10.1016/J.SOCSCIMED.2004.08.011 15652693

[bibr59-13634615241296302] Karasz A. Gany F. Escobar J. Flores C. Prasad L. Inman A. Kalasapudi V. Kosi R. Murthy M. Leng J. Diwan S. (2019). Mental health and stress among South Asians. Journal of Immigrant and Minority Health, 21(Suppl 1), 7–14. 10.1007/s10903-016-0501-4 27848078 PMC5643212

[bibr60-13634615241296302] Karasz A. Patel V. Kabita M. Shimu P. (2013). “Tension” in South Asian women: Developing a measure of common mental disorder using participatory methods. Progress in Community Health Partnerships: Research, Education, and Action, 7(4), 429–441. 10.1353/cpr.2013.0046 24375184 PMC4552248

[bibr61-13634615241296302] Kateri E. V. Tsouvelas G. Karademas E. C. (2019). The role of acculturation attitudes and social support in anxiety and depression of Indian immigrants in Greece. Psychiatrike = Psychiatriki, 30(4), 311-319, https://doi.org/10.22365/jpsych.2019.304.311. 10.22365/jpsych.2019.304.311 32283534

[bibr62-13634615241296302] Kendrick T. Pilling S. (2012). Common mental health disorders—identification and pathways to care: NICE clinical guideline. The British Journal of General Practice, 62(594), 47. 10.3399/BJGP12X616481 22520681 PMC3252532

[bibr63-13634615241296302] Keynejad R. (2011). Spirituality and mental health: Focus groups with faith leaders in a religiously diverse London borough. King's College.

[bibr64-13634615241296302] Kim S. B. Lee Y. J. (2021). Factors associated with mental health help-seeking among Asian Americans: A systematic review. Journal of Racial and Ethnic Health Disparities, 9(4), 1276–1297. 10.1007/S40615-021-01068-7/TABLES/2 34076864 PMC8170060

[bibr65-13634615241296302] Kleinman A. Eisenberg L. Good B. (2006). Culture, illness, and care: Clinical lessons from anthropologic and cross-cultural research. Focus, 4(1), 140–149. 10.1176/FOC.4.1.140 626456

[bibr66-13634615241296302] Knifton L. (2012). Understanding and addressing the stigma of mental illness with ethnic minority communities. Health Sociology Review, 21(3), 287–298. 10.5172/HESR.2012.21.3.287

[bibr67-13634615241296302] Kumari N. (2004). South Asian women in Britain: Their mental health needs and views of services. Journal of Public Mental Health, 3(1), 30–38. 10.1108/17465729200400005

[bibr68-13634615241296302] Lai D. W. L. Surood S. (2013). Effect of service barriers on health status of aging South Asian immigrants in Calgary, Canada. Health and Social Work, 38(1), 41–50. 10.1093/hsw/hls065 23539895

[bibr69-13634615241296302] Lavendera H. Khondoker A. H. Jones R. (2006). Understandings of depression: An interview study of Yoruba, Bangladeshi and White British people. Family Practice, 23(6), 651–658. 10.1093/fampra/cml043 16877452

[bibr70-13634615241296302] Lawrence V. Banerjee S. Bhugra D. Sangha K. Turner S. Murray J. (2006a). Coping with depression in later life: A qualitative study of help-seeking in three ethnic groups. Psychological Medicine, 36(10), 1375–1383. 10.1017/S0033291706008117 16854247

[bibr71-13634615241296302] Lawrence V. Murray J. Banerjee S. Turner S. Sangha K. Byng R. Bhugra D. Huxley P. Tylee A. Macdonald A. (2006b). Concepts and causation of depression: A cross-cultural study of the beliefs of older adults. The Gerontologist, 46(1), 23–32. http://gerontologist.oxfordjournals.org/ https://doi.org/10.1093/geront/46.1.23 16452281 10.1093/geront/46.1.23

[bibr72-13634615241296302] Lilhare V. K. Pathak A. Mathew K. Subudhi C. (2020). Explanatory model of mental illness and treatment-seeking behavior among caregivers of patients with mental illness: Evidence from Eastern India. Indian Journal of Social Psychiatry, 36(4), 327. 10.4103/IJSP.IJSP_24_20

[bibr73-13634615241296302] Loewenthal D. Mohamed A. Mukhopadhyay S. Ganesh K. Thomas R. (2012). Reducing the barriers to accessing psychological therapies for Bengali, Urdu, Tamil and Somali communities in the UK: Some implications for training, policy and practice. British Journal of Guidance and Counselling, 40(1), 43–66. 10.1080/03069885.2011.621519

[bibr74-13634615241296302] Lu J. Jamani S. Benjamen J. Agbata E. Magwood O. Pottie K. (2020). Global mental health and services for migrants in primary care settings in high-income countries: A scoping review. International Journal of Environmental Research and Public Health, 17(22), 1–28. 10.3390/IJERPH17228627 PMC7699722 33233666

[bibr75-13634615241296302] Lubin M. Khandai A. C. (2017). Prevalence and determinants of psychiatric disorders among South Asians in America. https://doi.org/10.1176/APPI.AJP-RJ.2016.110203, 11(2), 6–9. 10.1176/APPI.AJP-RJ.2016.110203

[bibr76-13634615241296302] Mallinson S. Popay J. (2007). Describing depression: Ethnicity and the use of somatic imagery in accounts of mental distress. Sociology of Health and Illness, 29(6), 857–871. 10.1111/j.1467-9566.2007.01048.x 17986019

[bibr77-13634615241296302] Markova V. Sandal G. M. Guribye E. (2020a). What do immigrants from various cultures think is the best way to cope with depression? Introducing the cross-cultural coping inventory. Frontiers in Psychology, 11, 1–17. 10.3389/fpsyg.2020.01599 PMC7372913 32760328

[bibr78-13634615241296302] Markova V. Sandal G. M. Pallesen S. (2020b). Immigration, acculturation, and preferred help-seeking sources for depression: Comparison of five ethnic groups. BMC Health Services Research, 20(1), 1–11. 10.1186/s12913-020-05478-x PMC7353801 32652988

[bibr79-13634615241296302] Masood N. Okazaki S. Takeuchi D. T. (2009). Gender, family, and community correlates of mental health in South Asian Americans. Cultural Diversity and Ethnic Minority Psychology, 15(3), 265–274. 10.1037/a0014301 19594255 PMC3955879

[bibr80-13634615241296302] Mathias K. Jain S. Fraser R. Davis M. Kimijima–Dennemeyer R. Pillai P. Deshpande S. N. Wolters M. (2023). Improving mental ill-health with psycho-social group interventions in South Asia – A scoping review using a realist lens. PLOS Global Public Health, 3(8), e0001736. 10.1371/JOURNAL.PGPH.0001736 PMC10461838 37639400

[bibr81-13634615241296302] McClelland A. Khanam S. Furnham A. (2014). Cultural and age differences in beliefs about depression: British Bangladeshis vs. British whites. Mental Health, Religion and Culture, 17(3), 225–238. 10.1080/13674676.2013.785710 PMC4095938 25076835

[bibr82-13634615241296302] Mokkarala S. Obrien E. K. Siegel J. T. (2016). The relationship between shame and perceived biological origins of mental illness among South Asian and white American young adults. Psychology, Health & Medicine, 21(4), 448–459. 10.1080/13548506.2015.1090615 26459610

[bibr83-13634615241296302] Naeem F. Khan T. Fung K. Narasiah L. Guzder J. Kirmayer L. J. (2020). Need to culturally adapt and improve access to evidence-based psychosocial interventions for Canadian South-Asians: A call to action. Canadian Journal of Community Mental Health, 38(4), 19–29. 10.7870/CJCMH-2019-016 .

[bibr84-13634615241296302] Page M. J. McKenzie J. E. Bossuyt P. M. Boutron I. Hoffmann T. C. Mulrow C. D. Shamseer L. Tetzlaff J. M. Akl E. A. Brennan S. E. Chou R. Glanville J. Grimshaw J. M. Hróbjartsson A. Lalu M. M. Li T. Loder E. W. Mayo-Wilson E. McDonald S. , …, Moher D. (2021). The PRISMA 2020 statement: An updated guideline for reporting systematic reviews. The BMJ, 372, 71. 10.1136/BMJ.N71 PMC8005924 33782057

[bibr85-13634615241296302] Patel V. Pereira J. Mann A. H. (1998). Somatic and psychological models of common mental disorder in primary care in India. Psychological Medicine, 28(1), 135–143. 10.1017/S0033291797005941 9483689

[bibr86-13634615241296302] Pollard T. Howard N. (2021). Mental healthcare for asylum-seekers and refugees residing in the United Kingdom: A scoping review of policies, barriers, and enablers. International Journal of Mental Health Systems, 15(1), 1–15. 10.1186/S13033-021-00473-Z/TABLES/3 34127043 PMC8201739

[bibr87-13634615241296302] Popay J. Roberts H. Sowden A. Petticrew M. Arai L. Rodgers M. Britten N.A7Roen K. Duffy S. (2006). Guidance on the conduct of narrative synthesis in systematic reviews: A product from the ESRC Methods Programme Peninsula Medical School, Universities of Exeter and Plymouth. Journal of Epidemiology and Community Health, 59(Suppl 1), A7. http://jech.bmj.com/content/vol59/suppl_1/

[bibr88-13634615241296302] Prajapati R. Liebling H. (2022). Accessing mental health services: A systematic review and meta-ethnography of the experiences of South Asian service users in the UK. Journal of Racial and Ethnic Health Disparities, 9(2), 598–619. 10.1007/S40615-021-00993-X 33686621 PMC8897382

[bibr89-13634615241296302] Rafique Z. (2010). An exploration of the presence and content of metacognitive beliefs about depressive rumination in Pakistani women. British Journal of Clinical Psychology, 49(3), 387–411. 10.1348/014466509X472020 19857378

[bibr90-13634615241296302] Rastogi P. Khushalani S. Dhawan S. Goga J. Hemanth N. Kosi R. Sharma R. K. Black B. S. Jayaram G. Rao V. (2014). Understanding clinician perception of common presentations in South Asians seeking mental health treatment and determining barriers and facilitators to treatment. Asian Journal of Psychiatry, 7(1), 15–21. 10.1016/J.AJP.2013.09.005 24524704

[bibr91-13634615241296302] Rathod S. Pinninti N. Irfan M. Gorczynski P. Rathod P. Gega L. Naeem F. (2017). Mental health service provision in low- and middle-income countries. Health Services Insights, 10, 1–7. 10.1177/1178632917694350 PMC5398308 28469456

[bibr92-13634615241296302] Roberts L. R. Mann S. K. Montgomery S. B. (2015). Depression, a hidden mental health disparity in an Asian Indian immigrant community. International Journal of Environmental Research and Public Health, 13(1), 1–17. 10.3390/ijerph13010027 PMC4730418 26703654

[bibr93-13634615241296302] Rüdell K. Bhui K. Priebe S. (2008). Do “alternative” help-seeking strategies affect primary care service use? A survey of help-seeking for mental distress. BMC Public Health, 8(207). 10.1186/1471-2458-8-207 PMC2443800 18547400

[bibr94-13634615241296302] Rutter M. Tienda M. (Eds.). (2005). Ethnicity and Causal Mechanisms. Cambridge University Press. https://doi.org/10.1017/CBO9781139140348

[bibr95-13634615241296302] Sangar M. Howe J. (2021). How discourses of sharam (shame) and mental health influence the help-seeking behaviours of British born girls of South Asian heritage. Educational Psychology in Practice, 37(4), 343–361. 10.1080/02667363.2021.1951676

[bibr96-13634615241296302] Segal U. A. (2018). Cultural variables in Asian Indian families. Families in Society, 72(4), 233–242. 10.1177/104438949107200406

[bibr97-13634615241296302] Shah M. Roy S. Ahluwalia A. (2023). Time to address the mental health challenges of the South Asian diaspora. *The Lancet Psychiatry,* *10*(6), 381-382, 10(6), 381–382. 10.1016/S2215-0366(23)00144-X 37208111

[bibr98-13634615241296302] Shariff A. (2009). Ethnic identity and parenting stress in South Asian families: Implications for culturally sensitive counselling. Canadian Journal of Counselling and Psychotherapy, 43(1), 35. https://cjc-rcc.ucalgary.ca/article/view/58908

[bibr99-13634615241296302] Sharma N. Shaligram D. Yoon G. H. (2020). Engaging South Asian youth and families: A clinical review. International Journal of Social Psychiatry, 66(6), 584–592. 10.1177/0020764020922881 32449476

[bibr100-13634615241296302] Sheikh S. Furnham A. (2000). A cross-cultural study of mental health beliefs and attitudes towards seeking professional help. Social Psychiatry and Psychiatric Epidemiology, 35(7), 326–334. 10.1007/S001270050246 11016528

[bibr101-13634615241296302] Singla D. R. MacKinnon D. P. Fuhr D. C. Sikander S. Rahman A. Patel V. (2021). Multiple mediation analysis of the peer-delivered thinking healthy programme for perinatal depression: Findings from two parallel, randomised controlled trials. The British Journal of Psychiatry, 218(3), 143–150. 10.1192/BJP.2019.184 31362799

[bibr102-13634615241296302] Soorkia R. Snelgar R. Swami V. (2011). Factors influencing attitudes towards seeking professional psychological help among South Asian students in Britain. Mental Health, Religion and Culture, 14(6), 613–623. 10.1080/13674676.2010.494176

[bibr103-13634615241296302] Steel Z. Marnane C. Iranpour C. Chey T. Jackson J. W. Patel V. Silove D. (2014). The global prevalence of common mental disorders: A systematic review and meta-analysis 1980–2013. International Journal of Epidemiology, 43(2), 476. 10.1093/IJE/DYU038 24648481 PMC3997379

[bibr104-13634615241296302] Taylor R. Brown J. S. L. Weinman J. (2013). A comparison of the illness perceptions of north Indian and white British women. Journal of Mental Health, 22(1), 22–32. 10.3109/09638237.2012.734664 23343044

[bibr105-13634615241296302] Tribe R. Marshall C. (2020). Culture, mental health, and immigrants. In R. Moodley & E. Lee (Eds.), The Routledge international handbook of race, culture and mental health (pp. 314–325). Routledge/Taylor & Francis Group.

[bibr106-13634615241296302] Tummala-Narra P. (2013). Psychotherapy with South Asian women: Dilemmas of the immigrant and first generations. Women and Therapy, 36(3–4), 176–197. 10.1080/02703149.2013.797853

[bibr107-13634615241296302] United Nations Department of Economic and Social Affairs. (2020). International migration 2020 highlights. United Nations. https://www.un.org/en/desa/international-migration-2020-highlights

[bibr108-13634615241296302] Wittkowski A. Zumla A. Glendenning S. Fox J. R. E. (2012). The experience of postnatal depression in South Asian mothers living in Great Britain: A qualitative study. Journal of Reproductive and Infant Psychology, 29(5), 480–492. 10.1080/02646838.2011.639014

