# Supplementary material for: Explanatory models of common mental disorders among South Asians in high-income countries: A systematic review
Source: Transcult Psychiatry. 2025 Jan 9;62(2):241–64. doi: 10.1177/13634615241296302 (PMC12130613; doi:10.1177/13634615241296302)
Supplement: sj-docx-1-tps-10.1177_13634615241296302 - Supplemental material for Explanatory models of common mental disorders among South Asians in high-income countries: A systematic review [file sj-docx-1-tps-10.1177_13634615241296302.docx]

**Explanatory Models of Common Mental Disorders among South Asians in High-Income Countries: A Systematic Review**

### **Supplementary Materials**

1. Search Strings

We searched the various databases using search terms under the following concepts: South Asian (e.g., Indian) AND Mental health problems (e.g., depression) AND explanatory models (e.g., beliefs) AND high-income countries (e.g., developed nation).

SA* or India* or Bangladesh* or Pakistan* or Sri Lanka* or Punjabi or Bengali or Gujarati or Asian American

**AND**
mental illness or mental health or mental disorder* or anxious or anxiet* or depress* or post-traumatic stress or PTSD or obsessive-compulsive disorder or OCD or panic disorder* or panic attack* or phobia* or generalised anxiety disorder* or GAD or wellbeing or well-being or emotional* distress* or psychological* distress* **OR** mental health services or counselling or counseling or therap* or psychological service* or psychotherapy

**AND**

Explanatory model* or crosscultur* or cultur* or conceptual* or understand* or percept* or belief* or attitud* or opinion* or knowledg* or idea or ideas or feeling or idiom*

**AND**

**(List of high-income countries) OR high-income country or high income country or high-income nation* or high income nation* or developed countr* or developed nation* or global north or Western countr* or Western nation*

***In the databases Medline and Embase, the HIC filter was applied. For the other databases, the list of HICs from Medline was copied and added as search line(s).*

1. SPIDER Framework

The SPIDER framework, adapted from the PICO search tool, was chosen because its components are better suited for qualitative and mixed-methods systematic reviews.

Table 2. SPIDER framework for inclusion/exclusion criteria.

|  | Inclusion criteria | Exclusion criteria (add any exclusion criteria that apply within each of the domains below) |
| --- | --- | --- |
| Sample | Participants that self-identify as a person of SA origin (from India, Pakistan, Bangladesh, and Sri Lanka); living in high-income countries (as defined by database search filters when applicable or the World Bank (add hyperlink)) | Studies that do not disaggregate results or discussion by the SA group as a whole or by sub-groups. |
| Phenomenon of interest | The influence of cultural context on mental health and mental health service utilisation |  |
| Design | Any qualitative, quantitative or mixed-method study design |  |
| Evaluation | Explanatory models of CMDs (depression and anxiety disorders) where explanatory models will be defined as “prior knowledge on the causation, perception, experiences, and traditional belief held by the patients, their caregiver, and the population in general.” (50). As defined by NICE Clinical Guidance, common mental health disorders are depression and anxiety disorders such as post-traumatic stress disorder, panic disorder, generalised anxiety disorder, obsessive-compulsive disorder, and phobias (49). |  |
|  | Help-seeking intentions, attitudes, behaviours/ mental health support use/help-seeking encounters for CMDs, where help-seeking will be defined as: “attempts to maximise wellness or to ameliorate, mitigate, or eliminate distress.” (51). Mental health services included will be broad in scope: informal (personal networks such as friends and family), formal (including professionals such as GPs, nurses, psychiatrists, and non-health professionals such as teachers, spiritual and religious leaders, and community workers) and self-help (“use of support available online”) (52)  **Help-seeking and associated terms were not included in the search strategy as they yielded too many irrelevant results; this criterion will be applied during title/abstract screening.* | Studies that solely explore the structural influences/aspects of understanding of mental health and service use (i.e., does not explore cultural context as a research aim) |
| Research type | Original, peer-reviewed research articles |  |
| **Other** |  |  |
| Language | Studies published in English | Studies not published in English due to the primary author’s language restrictions |
| Date of publication | Any |  |

1. List of Included Studies

Antoniades J, Mazza D, Brijnath B. Agency, activation and compatriots: the influence of social networks on health-seeking behaviours among Sri Lankan migrants and Anglo-Australians with depression. Sociol Health Illn. 2018 Nov 1;40(8):1376–90.

Antoniades J, Mazza D, Brijnath B. Becoming a patient-illness representations of depression of Anglo-Australian and Sri Lankan patients through the lens of Leventhal’s illness representational model. International Journal of Social Psychiatry. 2017 Nov 1;63(7):569–79.

Bhui K, Bhugra D, Goldberg D, Dunn G, Desai M. Cultural influences on the prevalence of common mental disorder, general practitioners’ assessments and help-seeking among Punjabi and English people visiting their general practitioner. Vol. 31, Psychological Medicine. 2001.

Bhui K, Bhugra D, Goldberg D. Causal explanations of distress and general practitioners’ assessments of common mental disorder among Punjabi and English attendees. Social Psychiatry and Psychiatric Epidemiology . 2002;37:38–45.

Bhui K, Rüdell K, Priebe S. Assessing Explanatory Models for Common Mental Disorders. J Clinical Psychiatry. 2006;67:964–71.

Birtel MD, Mitchell BL. Cross-cultural differences in depression between White British and South Asians: Causal attributions, stigma by association, discriminatory potential. Psychology and Psychotherapy: Theory, Research and Practice. 2023 Mar 1;96(1):101–16.

Brijnath B, Antoniades J. What is at stake? Exploring the moral experience of stigma with Indian-Australians and Anglo-Australians living with depression. Transcult Psychiatry. 2018 Apr 1;55(2):178–97.

Burr J, Chapman T. Contextualising experiences of depression in women from South Asian communities: a discursive approach. Vol. 26, Sociology of Health & Illness. 2004.

Chiu M, Amartey A, Wang X, Kurdyak P. Ethnic Differences in Mental Health Status and Service Utilization: A Population-Based Study in Ontario, Canada. Canadian Journal of Psychiatry. 2018 Jul 1;63(7):481–91.

Commander MJ, Odell SM, Surtees PG, Sashidharan SP. Care pathways for south Asian and white people with depressive and anxiety disorders in the community. Soc Psychiatry Psychiatr Epidemiol. 2004 Apr;39(4):259–64.

Ekanayake S, Ahmad F, McKenzie K. Qualitative cross-sectional study of the perceived causes of depression in South Asian origin women in Toronto. BMJ Open. 2012;2(1).

Farver JAM, Narang SK, Bhadha BR. East meets west: Ethnic identity, acculturation, and conflict in Asian Indian families. Journal of Family Psychology. 2002;16(3):338–50.

Fernández de la Cruz L, Kolvenbach S, Vidal-Ribas P, Jassi A, Llorens M, Patel N, et al. Illness perception, help-seeking attitudes, and knowledge related to obsessive–compulsive disorder across different ethnic groups: a community survey. Soc Psychiatry Psychiatr Epidemiol. 2016 Mar 1;51(3):455–64.

Furnham A, Malik R. Cross-Cultural Beliefs About Depression. International Journal of Social Psychiatry . 1994;40(2):126–33.

Gask L, Aseem S, Waquas A, Waheed W. Isolation, feeling “stuck” and loss of control: Understanding persistence of depression in British Pakistani women. J Affect Disord. 2011 Jan;128(1–2):49–55.

Gilbert AS, Antoniades J, Bowen Z, Brijnath B. Legitimising depression: community perspectives and the help-seeking continuum. Health Sociology Review. 2019 Sep 2;28(3):291–306.

Hanley J. The emotional wellbeing of Bangladeshi mothers during the postnatal period. Community Practitioner . 2007;80(5):34–7.

Jacob KS, Bhugra D, Lloyd KR, Mrcpsych1 M, Mann AH. Common mental disorders, explanatory models and consultation behaviour among Indian women living in the UK. J R Soc Med. 1998;91:66–71.

Kateri E V, Tsouvelas G, Karademas EC. The role of acculturation attitudes and social support in anxiety and depression of Indian immigrants in Greece. Vol. 30, PSYCHIATRIKI. 2019.

Kumari N. South Asian women in Britain: their mental health needs and views of services. Journal of Mental Health Promotion. 2004;3(1):30–8.

Lavendera H, Khondoker AH, Jones R. Understandings of depression: An interview study of Yoruba, Bangladeshi and White British people. Fam Pract. 2006 Dec 1;23(6):651–8.

Lawrence V, Banerjee S, Bhugra D, Sangha K, Turner S, Murray J. Coping with depression in later life: A qualitative study of help-seeking in three ethnic groups. Psychol Med. 2006 Oct;36(10):1375–83.

Lawrence V, Murray J, Banerjee S, Turner S, Sangha K, Byng R, et al. Concepts and Causation of Depression: A Cross-Cultural Study of the Beliefs of Older Adults. Gerontologist [Internet]. 2006;46(1):23–32. Available from: <http://gerontologist.oxfordjournals.org/>

Loewenthal D, Mohamed A, Mukhopadhyay S, Ganesh K, Thomas R. Reducing the barriers to accessing psychological therapies for Bengali, Urdu, Tamil and Somali communities in the UK: Some implications for training, policy and practice. Br J Guid Counc. 2012 Feb;40(1):43–66.

Mallinson S, Popay J. Describing depression: Ethnicity and the use of somatic imagery in accounts of mental distress. Sociol Health Illn. 2007 Sep;29(6):857–71.

Markova V, Sandal GM, Guribye E. What Do Immigrants From Various Cultures Think Is the Best Way to Cope With Depression? Introducing the Cross-Cultural Coping Inventory. Front Psychol. 2020 Jul 14;11.

Markova V, Sandal GM, Pallesen S. Immigration, acculturation, and preferred help-seeking sources for depression: Comparison of five ethnic groups. BMC Health Serv Res. 2020 Jul 11;20(1).

McClelland A, Khanam S, Furnham A. Cultural and age differences in beliefs about depression: British Bangladeshis vs. British Whites. Ment Health Relig Cult. 2014 Mar;17(3):225–38.

Rafique Z. An exploration of the presence and content of metacognitive beliefs about depressive rumination in Pakistani women. British Journal of Clinical Psychology. 2010 Sep;49(3):387–411.

Roberts LR, Mann SK, Montgomery SB. Depression, a hidden mental health disparity in an Asian Indian immigrant community. Int J Environ Res Public Health. 2015 Dec 23;13(1).

Rüdell K, Bhui K, Priebe S. Do “alternative” help-seeking strategies affect primary care service use? A survey of help-seeking for mental distress. BMC Public Health. 2008;8.

Taylor R, Brown JSL, Weinman J. A comparison of the illness perceptions of North Indian and white British women. Journal of Mental Health. 2013 Feb;22(1):22–32.

Wittkowski A, Zumla A, Glendenning S, Fox JRE. The experience of postnatal depression in South Asian mothers living in Great Britain: A qualitative study. J Reprod Infant Psychol. 2012 Nov;29(5):480–92.

1. Quality Assessment

Details of quality assessment for the included articles.

Table 3. MMAT for Assessing Quantitative Studies.

| **Authors**  **Year** | **Are there clear research questions?** | **Do the collected data allow to address the research questions?** | **Is the sampling strategy relevant to address the research question?** | **Is the sample representative of the target population?** | **Are the measurements appropriate?** | **Is the risk of nonresponse bias low?** | **Is the statistical analysis appropriate to answer the research question?** |
| --- | --- | --- | --- | --- | --- | --- | --- |
| Furnam and Malik 1994 | Y | Y | Y | Y | Y | Y | Y |
| Bhui et al.  2001, 2002 | Y | Y | Y | Y | Y | Y | Y |
| Faver et al.  2002 | Y | Y | Y | Y | Y | Y | Y |
| Kumari  2004 | N | M | Y | Y | Y | Y | M |
| Bhui et al.  2006 | Y | Y | Y | Y | Y | Y | Y |
| Taylor et al.  2013 | Y | Y | Y | Y | Y | Y | Y |
| McClelland et al.  2015 | Y | Y | Y | Y | Y | Y | Y |
| de la Cruz et al.  2016 | Y | Y | Y | Y | Y | Y | Y |
| Chiu et al.  2018 | Y | Y | Y | Y | Y | Y | Y |
| Kateri et al.  2019 | Y | Y | Y | Y | Y | Y | Y |
| Markova et al.  2020 | Y | Y | Y | Y | Y | Y | Y |

Table 4. CASP Checklist for Assessing Qualitative Studies.

| **Authors**  **Year** | **Was there a clear statement of the aims of the research?** | **Is a qualitative methodology appropriate?** | **Was the research design appropriate to address the aims of the research?** | **Was the recruitment strategy appropriate to the aims of the research?** | **Was the data collected in a way that addressed the research issue?** | **Has the relationship between researcher and participants been adequately considered?** | **Have ethical issues been taken into consideration?** | **Was the data analysis sufficiently rigorous?** | **Is there a clear statement of findings?** | **How valuable is the research?** |
| --- | --- | --- | --- | --- | --- | --- | --- | --- | --- | --- |
| Burr and Chapman  2004 | Y | Y | Y | Y | Y | Y | Y | Y | Y | Valuable |
| Lavendera et al.  2006 | Y | Y | Y | Y | Y | Y | Y | Y | Y | Valuable |
| Lawrence et al.  2006 | Y | Y | Y | Y | Y | N | Y | M | Y | Valuable |
| Hanley  2007 | Y | Y | Y | Y | Y | Y | Y | Y | Y | Valuable |
| Mallinson and Popay  2007 | Y | Y | Y | Y | Y | Y | Y | Y | Y | Valuable |
| Gask et al.  2010 | Y | Y | Y | Y | Y | Y | Y | Y | Y | Valuable |
| Rafique  2010 | Y | Y | Y | Y | Y | Y | Y | Y | Y | Valuable |
| Loewenthal et al.  2011 | Y | Y | Y | Y | Y | Y | Y | Y | Y | Valuable |
| Wittkowski et al.  2011 | Y | Y | Y | Y | Y | Y | Y | Y | Y | Valuable |
| Ekanayake et al.  2012 | Y | Y | Y | Y | Y | Y | Y | Y | Y | Valuable |
| Antoniades et al.  2017, 2018 | Y | Y | Y | Y | Y | N | Y | Y | Y | Valuable |
| Brijnath and Antoniades  2018 | Y | Y | Y | M | Y | N | Y | M | Y | Valuable |
| Gilbert et al.  2019 | Y | Y | Y | M | Y | N | Y | Y | Y | Valuable |
| Birtel and Mitchell 2023 | Y | Y | Y | Y | Y | N | Y | Y | Y | Valuable |

Table 4. MMAT for Mixed Methods Studies.

| **Authors**  **Year** | **Is there an adequate rationale for using a mixed methods design to address the research question?** | **Are the different components of the study effectively integrated to answer the research question?** | **Are the outputs of the integration of qualitative and quantitative components adequately interpreted?** | **Are divergences and inconsistencies between quantitative and qualitative results adequately addressed?** | **Do the different components of the study adhere to the quality criteria of each tradition of the methods involved?** |
| --- | --- | --- | --- | --- | --- |
| Jacob et al.  1998 | Y | Y | Y | Y | Y |
| Commander et al.  2003 | Y | Y | Y | Y | Y |
| Rudell et al.  2008 | Y | Y | Y | Y | Y |
| Roberts et al.  2015 | Y | Y | Y | Y | Y |
